# Development of a 12-item short version of the HIV stigma scale

**DOI:** 10.1186/s12955-017-0691-z

**Published:** 2017-05-30

**Authors:** Maria Reinius, Lena Wettergren, Maria Wiklander, Veronica Svedhem, Anna Mia Ekström, Lars E. Eriksson

**Affiliations:** 1 Department of Learning, Informatics, Management and Ethics, Karolinska Institutet, SE-171 77 Stockholm, Sweden; 2 Department of Neurobiology, Care Sciences and Society, Karolinska Institutet, SE-141 83 Stockholm, Sweden; 3 Unit of Infectious Diseases, Department of Medicine Huddinge, Karolinska Institutet, SE-141 83 Stockholm, Sweden; 4 Department of Infectious Diseases, Karolinska University Hospital, SE-141 86 Stockholm, Sweden; 5 Department of Public Health, Karolinska Institutet, SE-171 77 Stockholm, Sweden; 6 School of Health Sciences, City, University of London, EC1V 0HB, London, UK

**Keywords:** HIV, Instrument, Nursing, Patient-reported outcome measures, Psychometrics, Short-form, Stigma

## Abstract

**Background:**

Valid and reliable instruments for the measurement of enacted, anticipated and internalised stigma in people living with HIV are crucial for mapping trends in the prevalence of HIV-related stigma and tracking the effectiveness of stigma-reducing interventions. Although longer instruments exist, e.g., the commonly used 40-item HIV Stigma Scale by Berger et al., a shorter instrument would be preferable to facilitate the inclusion of HIV stigma in more and broader surveys. Therefore, the aim of this work was to develop a substantially shorter, but still valid, version of the HIV Stigma Scale.

**Methods:**

Data from a psychometric evaluation of the Swedish 40-item HIV Stigma Scale were reanalysed to create a short version with 12 items (three from each of the four stigma subscales: *personalised stigma*, *disclosure concerns*, *concerns with public attitudes* and *negative self-image*). The short version of the HIV stigma scale was then psychometrically tested using data from a national survey investigating stigma and quality of life among people living with HIV in Sweden (*n* = 880, mean age 47.9 years, 26% female).

**Results:**

The hypothesized factor structure of the proposed short version was replicated in exploratory factor analysis without cross loadings and confirmatory factor analysis supported construct validity with high standardised effects (>0.7) of items on the intended scales. The χ^2^ test was statistically significant (χ^2^ = 154.2, df = 48, *p* < 0.001), but alternate fit measures indicated acceptable fit (comparative fit index: 0.963, Tucker-Lewis index: 0.950 and root mean square error of approximation: 0.071). Corrected item-total correlation coefficients were >0.4 for all items, with a variation indicating that the broadness of the concept of stigma had been captured. All but two aspects of HIV-related stigma that the instrument is intended to cover were captured by the selected items in the short version. The aspects that did not lose any items were judged to have acceptable psychometric properties. The short version of the instrument showed higher floor and ceiling effects than the full-length scale, indicating a loss of sensitivity in the short version. Cronbach’s α for the subscales were all >0.7.

**Conclusions:**

Although being less sensitive in measurement, the proposed 12-item short version of the HIV Stigma Scale has comparable psychometric properties to the full-length scale and may be used when a shorter instrument is needed.

## Introduction

HIV-related stigma is prevalent in many parts of the world and affects the quality of life of people living with HIV [1–4]. HIV-related stigma is also a common barrier to HIV testing and treatment adherence [5–10]. Valid and reliable instruments for the measurement of enacted, anticipated and internalised stigma in people living with HIV are crucial for mapping trends in the prevalence of HIV-related stigma [10]. There are several instruments designed to measure HIV stigma, where Berger et al.’s [11] 40-item HIV Stigma Scale is the most commonly used and is one of only a few instruments that cover all stigma mechanisms affecting people with HIV [10]. We recently adapted and validated this scale for the Swedish context and, after removing one item, a 39-item scale showed satisfactory construct validity and reliability [12]. The over-determination of the full-length scales, with high Cronbach’s α (0.883–0.958), indicated that the number of items could be reduced. Furthermore, the original 40-item scale may take up to 25 min to complete [13]. Shortened versions, which respectively cover 25 and 32 items of the HIV Stigma Scale, have been published previously [13, 14]. However, to facilitate the inclusion of HIV stigma in more extensive surveys, a shorter instrument would be preferable. Although short forms exist for children and young adults [15–17], beyond the abovementioned examples, no other shorter versions of the HIV Stigma Scale have been published for adults living with HIV. The aim of this work was therefore to develop a substantially shorter version of the HIV Stigma Scale with psychometric properties retained from the full-length scale.

## Methods

The short version of the HIV Stigma Scale was developed in two phases: 1) Our data from the validation of the 40-item HIV Stigma Scale in Sweden (*n* = 132, 55 female, 77 male, age 23–74; mean 48.3, SD 11.0) [12] were reanalysed in order to select items for a short version. 2) To ensure construct validity and reliability of the shorter version, psychometric analysis was also performed on data from an additional sample who had responded to our proposed short version of the HIV Stigma Scale.

### Phase 1. Item reduction

**Fig. 1:**
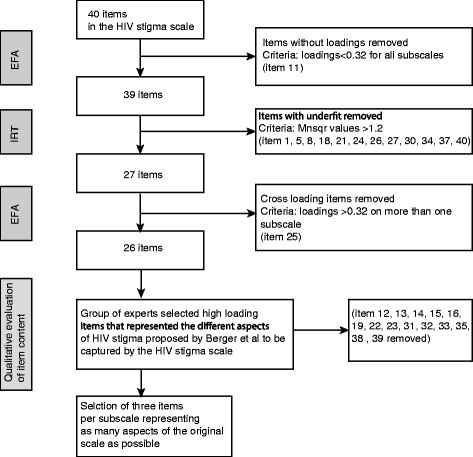
Flowchart over the item reduction process to form a short form version of the HIV Stigma Scale

The HIV Stigma Scale consists of four subscales intended to measure *personalised stigma*, *disclosure concerns*, c*oncerns with public attitudes* and *negative self-image* [11]. Berger et al. [11] described that each of these subscales contains between two and three main aspects, as summarised in Table 1. The intention when developing a short version of the HIV Stigma Scale was to select items in the instrument that showed good psychometric properties, but also to maintain as many as possible of the aspects of HIV stigma that the original instrument was intended to cover. The item reduction process is presented schematically in Fig. 1 and in more detail below.

**Table 1:** Main aspects of subscales and items selected for the short version of the HIV Stigma Scale

Subscale	Description of content excerpted from Berger et al.^a^	Interpretation of main aspects	Questions selected for the short version of the HIV Stigma Scale
Personalised stigma	‘…Perceived consequences of other people knowing that the respondent has HIV, such as losing friends, feeling that people were avoiding him/her, and regrets for having told people’.	1. Losing friends and fear of rejection.	29. People I care about stopped calling after learning I have HIV
			36. I have lost friends by telling them I have HIV
		2. Feeling that people avoid me.	28. Some people avoid touching me if they know I have HIV
		3. Regrets for having told people about my HIV status	All items regarding this aspect cross-loaded in analyses or had underfit
Disclosure concerns	‘…controlling information, keeping one’s HIV status a secret, or worrying that others who knew about respondent’s HIV status would tell’.	1. Keeping my HIV status a secret	4. Telling someone I have HIV is risky
			6. I work hard to keep my HIV a secret
			17. I am very careful who I tell that I have HIV
		2. Worrying that others will disclose my HIV status	All items regarding this aspect cross-loaded in the analyses or had underfit
Concerns about public attitudes	‘…what most people think about people with HIV or what most people with HIV can expect when others learn they have HIV … the consequences of people in general knowing about a person having HIV’.	1. What most people think about people with HIV	10. Most people believe a person who has HIV is dirty
			20. Most people are uncomfortable around someone with HIV
		2. Consequences of people in general knowing about a person having HIV	9. People with HIV are treated like outcasts
Negative self-image	‘…feeling unclean, not as good as others or like a bad person because of HIV … feelings of shame and guilt’.	1. Negative feelings, guilt, shame, feeling unclean	2. I feel guilty because I have HIV
		2. Feeling like I am a bad person because of HIV	7. I feel I’m not as good a person as others because I have HIV
			3. People’s attitudes about HIV make me feel worse about myself

^a^Berger BE, Ferrans CE, Lashley FR. Measuring stigma in people with HIV: psychometric assessment of the HIV stigma scale. Res Nurs Health. 2001;24(6);518–29 [11]

#### Step 1. Removing items with underfit

All items in the full-length scale were examined with item response theory methods to find items with underfit [18]. Partial credit models were calculated for each subscale separately using the package eRm [19] in R statistics [20] and item fit statistics were assessed. Items with outfit or infit mean square values exceeding 1.2 were considered to have underfit [18] and were not considered for the short version of the HIV stigma scale.

#### Step 2. Removing cross-loading items

Remaining items were evaluated regarding their loading in our previously published exploratory factor analysis performed on data from 132 persons living with HIV in Sweden [12]. Cross loading items were not considered for the short version of the HIV stigma scale.

#### Step 3. Keeping as many aspects as possible

A group of professionals working in academia and HIV care and with expertise in HIV and psychometrics discussed which of the remaining items best represented the different aspects of HIV stigma that Berger et al. [11] intended the instrument to cover. They agreed on three selected items from each of the four subscales to be included in the Phase 2 assessment of a tentative 12-item short version of the HIV Stigma Scale. The same response format from the original scale was used, i.e. a 4-point Likert scale, ranging from strongly disagree (1) to strongly agree (4). Responses were summed to calculate subscale scores with a possible range of 3 to 12; higher scores reflect a higher level of perceived HIV-related stigma.

### Phase 2. Psychometric evaluation of the short version of the HIV stigma scale

The proposed short version of the HIV Stigma Scale was distributed as part of a longer self-administered anonymous questionnaire to a sample of individuals participating in the nationwide study ‘Living with HIV in Sweden’ [21]. This nationwide study investigated the quality of life of people living with HIV in Sweden and was performed December 2013 through August 2014.

### Participants

The inclusion criteria were as follows: 1) >18 years of age and 2) having been diagnosed with HIV >6 months. Participants were recruited consecutively at 15 different centres for HIV care across Sweden, resulting in a total of 1096 valid responses (response rate ranging between 36 and 70% for different centres). The recruited sample was judged to be representative of people living with HIV in Sweden [21], where the WHO UNAIDS 90–90-90 goals are met, with 78% of the population of people living with HIV being virologically suppressed [22]. In December 2015, 6946 persons diagnosed with HIV in Sweden were linked to care, which corresponds to 99.8% of all persons diagnosed with HIV in Sweden. Of these, 95.1% were on antiretroviral therapy and 94.7% of those who had been on treatment for at least 6 months had a viral load <50 HIV-1 RNA copies/mL [22]. For the present analysis, a subsample of 880 questionnaires with complete answers to the 12-item HIV Stigma Scale was used (age range 18–82 years, mean age 47.9, 26% female).

### Construct validity

The sample of 880 completed questionnaires was randomly divided into two groups of equal size, where the first part was analysed in an exploratory factor analysis and the second part in confirmatory factor analysis, to ensure factor stability. The exploratory factor analysis was performed in SPSS 23 with alpha factoring, oblimin rotation. The confirmatory factor analysis model that represented the short version of the HIV Stigma Scale was then set up and analysed with maximum likelihood using the lavaan package [23] in R statistics [20]. Goodness of fit was evaluated using χ^2^ testing and was expected to be non-significant if the data had a good fit to the model; root mean square error of approximation (RMESA), where a score below 0.05 indicates good fit; and comparative fit index (CFI) and Tucker-Lewis index (TLI), with a desired value of >0.90 for both indexes [24]. Corrected item-total correlation coefficients were calculated for each item, which were expected to exceed 0.4 and also have a variation in range to ensure that the broadness of the measured concept had been captured by the short version of the scale [25]. Floor and ceiling effects were calculated and compared to the Swedish 39-item version of the HIV Stigma Scale (where less than 15% of participants had the lowest or highest possible score [12], which is considered acceptable [26]).

### Reliability

Cronbach’s α was assessed for the subscales to ensure internal consistency and was considered acceptable if >0.7 [27].

## Results

### Phase 1. Item reduction

Step 1. Removing items with underfit.

Twelve items showed underfit (Table 2) and were removed.

**Table 2:** Factor loadings^a^ and outfit/infit mean square measures^b^ for all items in the HIV stigma scale

Items	Component				Outfit Msqr	Infit Msqr
	1	2	3	4		
39. People seem afraid of me once they learn I have HIV	0.866				0.485	0.511
29. People I care about stopped calling after learning I have HIV	0.864				0.592	0.661
38. People who know I have HIV tend to ignore my good points	0.833				0.530	0.557
28. People avoid touching me once they know I have HIV	0.824				0.654	0.748
35. I have stopped socializing with some people due to their reaction to me having HIV	0.776				0.641	0.702
36. I have lost friends by telling them I have HIV	0.770				0.748	0.869
33. People have physically backed away from me when they learn I have HIV	0.726				0.729	0.735
24. I have been hurt by how people reacted to learning I have HIV	0.721				**1.337**	**1.267**
32. People don’t want me around their children once they know I have HIV	0.714				0.975	1.013
30. Some people told me that getting HIV is what I deserve for how I have lived my life	0.700				1.164	**1.383**
27. As a rule, telling others that I have HIV has been a mistake	0.677	0.344			1.159	**1.205**
31. Some people close to me are afraid others will reject them if it becomes known that I have HIV	0.671				1.093	1.101
26. I regret having told some people that I have HIV	0.642	0.327			**1.680**	**1.505**
34. Some people act as though it’s my fault I have HIV	0.638				**1.212**	0.881
40. When people learn you have HIV, they look for flaws in your character	0.627				**1.262**	0.978
18. Some people who know I have HIV have grown more distant	0.604				**1.999**	**1.456**
6. I work hard to keep my HIV a secret		0.751			0.580	0.627
17. I am very careful who I tell that I have HIV		0.746			0.637	0.686
1. In many areas of my life, no one knows I have HIV		0.696			**1.559**	1.142
21. I never feel I need to hide the fact I have HIV (R)		0.575	−0.411		**1.334**	**1.291**
4. Telling someone I have HIV is risky		0.614			0.870	0.804
25. I worry people who know I have HIV will tell others	0.362	0.542			0.841	0.858
22. I worry that people may judge me when they learn I have HIV		0.493			0.763	0.733
37. I have told people close to me to keep the fact that I have HIV a secret		0.402			**1.333**	**1.248**
15. Having HIV makes me feel that I’m a bad person			−0.737		0.667	0.725
7. I feel I’m not as good a person as others because I have HIV			-0.698		0.738	0.696
3. People’s attitudes about HIV make me feel worse about myself			−0.665		0.762	0.817
8. I never feel ashamed of having HIV (R)			-0.654		**1.361**	**1.406**
12. Having HIV makes me feel unclean			−0.570		0.778	0.790
2. I feel guilty because I have HIV			-0.532		1.105	1.022
23. Having HIV in my body is disgusting to me			−0.530		0.970	0.991
13. Since learning I have HIV, I feel set apart and isolated from the rest of the world	0.357		−0.484		0.983	0.967
20. Most people are uncomfortable around someone with HIV				-0.769	0.550	0.560
9. People with HIV are treated like outcasts				−0.639	1.016	0.941
14. Most people think that a person with HIV is disgusting				−0.613	0.865	0.806
10. Most people believe a person who has HIV is dirty				−0.599	0.734	0.760
16. Most people with HIV are rejected when others find out				−0.598	1.049	1.076
5. People with HIV lose their jobs when employers find out				−0.477	**1.243**	**1.206**
19. Since leardning I have HIV, I worry about people discriminating against me				−0.389	0.926	0.837
11. It is easier to avoid new friendship than worry about telling someone that I have HIV^c^						

^a^Factor component scores are reproduced under the creative common licence CC-BY from our previous work Lindberg MH, Wettergren L, Wiklander M, Svedhem-Johansson V, Eriksson LE. Psychometric Evaluation of the HIV Stigma Scale in a Swedish Context. PloS One. 2014;9(12):e114867 [12]. The analysis was performed on 132 completed questionnaires from persons living with HIV in Sweden

^b^Infit and outfit Meansquare values calculated through Partial credit models, Item response theory. Infit/outfit msqr values >1.2 were considered to have underfit (bold)

^c^Item 11 was removed from the Swedish version of the HIV stigma scale, due to low loadings on all factors, and was thus not included in the partial credit model

Step 2. Removing cross-loading items.

One of the remaining items, item 25, cross-loaded in the exploratory factor analysis (Table 2) and was removed.

Step 3. Keeping as many aspects as possible.

From the remaining items, three items were chosen from each subscale. We chose a selection of items that covered as many aspects of the concepts as possible. If more than one item covered an aspect, the item with highest loading was chosen. The selected items and the aspects they cover are shown in Table 1. For *personalised stigma* and *disclosure concerns*, one aspect was lost in each subscale due to cross-loading items or items with underfit (regrets that a person can have over disclosing one’s HIV status and worry that someone else will disclose one’s HIV status).

### Phase 2. Psychometric evaluation of the short version of the HIV Stigma Scale

#### Construct validity

**Fig. 2:**
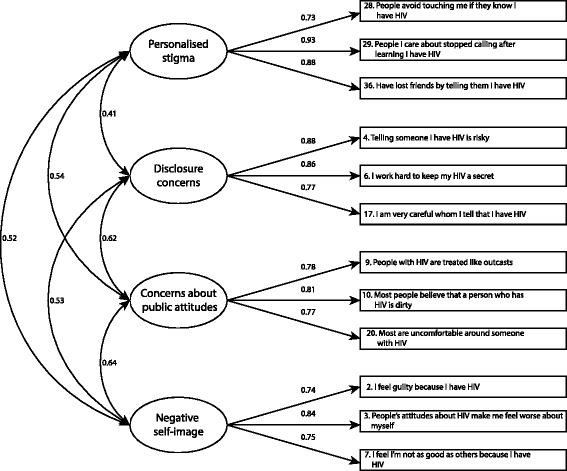
Confirmatory factor analysis of the short version of the HIV Stigma Scale. Results show correlations between subscales (*circles*) and maximum likelihood estimates for the relation between subscales and items (*squares*). The sample (*n* = 440) was randomly selected from all respondents with complete answers in a Swedish population of people living with HIV. Maximum likelihood estimates are standardised

In the exploratory factor analysis (alpha factoring, oblimin rotation) the factor structure suggested for the short version of the HIV stigma scale was replicated without cross loadings (Table 3). Eigenvalues for the four factors were 5.61, 1.50, 1.21 and 1.01 respectively. Results from the confirmatory factor analysis with standardised effects and correlation coefficients are presented in Fig. 2. Construct validity of the scale was supported with high standardised effects (>0.7) of items on the intended scales. The χ^2^ test was statistically significant (χ^2^ = 154.2, df = 48, *p* < 0.001), but the alternate fit measures indicated acceptable fit; CFI: 0.963; TLI: 0.950 and RMSEA: 0.071. Descriptive statistics for the scale are presented in Table 4 on the item level and subscale level. Corrected item-total correlation coefficients exceeded 0.4 for all items and had a variation in the range 0.62–0.84 (Table 4), indicating that the broadness of the intended stigma concepts had been captured. Floor or ceiling effects exceeded 15% for *Personalized stigma* (28% of participants had lowest possible score), *Disclosure concerns* (22% of participants had highest possible score and *Negative self-image* (24% of participants had lowest possible score (Table 4).

**Table 3:** Results from exploratory factor analysis^a^

	Factors^b^			
	1	2	3	4
Eigenvalues	5.61	1.50	1.21	1.01
Item				
Personalised stigma				
29. People I care about stopped calling after learning I have HIV	**0.978**	−0.010	−0.044	0.025
36. I have lost friends by telling them I have HIV	**0.862**	0.005	−0.081	−0.093
28. Some people avoid touching me once they know I have HIV	**0.614**	−0.005	0.245	0.012
Disclosure concerns				
6. I work hard to keep my HIV a secret	−0.025	**−0.870**	−0.054	−0.036
4. Telling someone I have HIV is risky	−0.017	**−0.764**	−0.041	−0.146
17. I am very careful who I tell that I have HIV	0.005	**−0.748**	0.135	0.086
Concerns about public attitudes				
10. Most people believe a person who has HIV is dirty	−0.023	−0.001	**0.783**	−0.103
9. People with HIV are treated like outcasts	0.035	0.023	**0.705**	−0.145
20. Most people are uncomfortable around someone with HIV	0.132	−0.260	**0.604**	0.094
Negative self-image				
2. I feel guilty because I have HIV	0.012	−0.041	−0.038	**−0.759**
3. People’s attitudes about HIV make me feel worse about myself	0.033	−0.046	0.046	**−0.758**
7. I feel I’m not as good a person as others because I have HIV	0.033	0.008	0.130	**−0.663**

^a^Alpha factoring, oblimin rotation on data from the study “Living with HIV in Sweden” (*n* = 440)

^b^Factor loadings <-0.32 or >0.32 in bold

**Table 4:** Descriptive statistics for items and subscales in the short-form version of the HIV Stigma Scale^a^

	Mean item score^b^ (SD)	Corrected item-total correlation	Mean subscale score^c^ (SD)	Reliability, α	Floor/ceiling effect (%)
Personalised stigma			6.09 (2.75)	0.88	28/6
28. Some people avoid touching me once they know I have HIV	2.06 (1.00)	0.70			
29. People I care about stopped calling after learning I have HIV	1.97 (1.00)	0.84			
36. I have lost friends by telling them I have HIV	2.06 (1.06)	0.78			
Disclosure concerns			9.08 (2.57)	0.84	6/22
4. Telling someone I have HIV is risky	2.97 (0.98)	0.68			
6. I work hard to keep my HIV a secret	2.97 (1.02)	0.74			
17. I am very careful who I tell that I have HIV	3.25 (0.96)	0.68			
Concerns about public attitudes			7.60 (2.50)	0.81	9/9
9. People with HIV are treated like outcasts	2.43 (0.97)	0.67			
10. Most people believe a person who has HIV is dirty	2.54 (0.99)	0.71			
20. Most people are uncomfortable around someone with HIV	2.64 (0.97)	0.62			
Negative self-image			6.40 (2.75)	0.80	24/5
2. I feel guilty because I have HIV	2.21 (1.09)	0.62			
3. People’s attitudes about HIV make me feel worse about myself	2.17 (1.07)	0.70			
7. I feel I’m not as good a person as others because I have HIV	2.01 (1.09)	0.62			

^a^Participants with complete answers, *n* = 880

^b^Possible score for each item 1–4; higher scores reflect a higher level of perceived HIV-related stigma

^c^Possible score 3–12 on each scale; higher scores reflect a higher level of perceived HIV-related stigma

^SD^Standard deviation

### Reliability

Cronbach’s α for the final combination of items for the subscales were all above 0.7 and considered acceptable (Table 4).

## Discussion

This report describes the development of a 12-item short version of the HIV Stigma Scale, that mainly preserves the broad concepts and internal consistency of the original subscales [11]. Since cross-loading items were excluded from the short version and no cross-loadings appeared in the exploratory factor analysis, we believe that this short version may have better psychometric properties than the original full length HIV Stigma Scale, which has exhibited overlap of items between several subscales [14]. The subscales in the short versions are highly intercorrelated, which reflects the psychometric properties of the original full length HIV stigma scale. However, a significant χ2 test of the confirmatory factor analysis indicated a misfit between the short version of the instrument and the data. Although it is known that even minor differences can generate a statistically significant χ2 value [28], we suggest further psychometric testing of the short version of the HIV stigma scale to examine whether signs of overlap of the variance between subscales will occur. Nevertheless, the other measures of model fit used, including standardised maximum likelihood estimates above 0.7 in the expected direction together with alternate fit measures within an acceptable range, supported the construct validity of the short scale in a Swedish context; whether this holds true for different populations remains to be investigated. The sample used for testing of the proposed short version had a high proportion of men, which reflects the gender distribution of persons living with HIV in Sweden. Specific gender related properties of the instrument can therefore have been missed and should be observed in future studies.

The exploratory factor analysis that formed the basis for item selection was performed on a relatively small sample (*n* = 132). However, the overdetermined factors, together with a wide range of communalities, supported reliability of the resulting solution [29]. When choosing between using the full length scale or the developed short form, the higher floor and ceiling effects shown for the short version should be taken into account; as expected, the short version is less sensitive in detecting different levels of perceived stigma. Although less sensitive than the full-length scale, we consider the proposed 12-item short version of the HIV Stigma Scale to have essentially the same psychometric properties as the full-length scale and propose that it may be used when a shorter instrument is desirable. The short version could, for example, be used if there is a wish to include a brief stigma component in longer surveys investigating the life situation of people living with HIV, in clinical contexts as a brief screening measure for signs of stigma-related problems or to serve as a basis for discussions with individual clients.

## Conclusions

Although being less sensitive in measurement, the proposed 12-item short version of the HIV Stigma Scale has comparable psychometric properties to the full-length scale and may be used when a shorter instrument is needed.

## Abbreviations

CFI: Comparative fit index
RMSEA: Root mean square error of approximation
TLI: Tucker-Lewis index
